# The Christian Origin of Hospitals

**Published:** 1890-02-08

**Authors:** 


### THE CHRISTIAN ORIGIN OF HOSPITALS.

THE care of the sick has been from the earliest times recognised as peculiarly a Christian duty. Without discussing the bearing of the New Testament upon the matter, that it was at once acknowledged as part of the routine work of the Church cannot be doubted. The Apostolic Constitutions (a collection of documents at least earlier than the Council of Nice, 325) define one of the chief duties of the deacons to be the visitation of the poor and the sick. In this work they were helped by the deaconesses, to whom would naturally be relegated the care of their own sex. Early in the second century Justin Martyr speaks of the deacon carrying the Holy Communion to the sick in their own homes. But in those primitive days no attempt seems to have been made to gather together those who were in need of care and attendance, and it would appear that houses for the reception of the poor and of travellers were established before those for the reception of the suffering. Equally with the care of the sick, hospitality was a great feature of primitive Christian life. In the earliest days Christians received travellers into their own homes, but with the progress of time this custom became both insufficient and liable to abuse. Accordingly houses were built under Christian auspices for the convenience of travellers, naturally in those spots and along those routes most frequented by Christian pilgrims. Anticipating for a moment—the name of the superintendent of one such hostelry has come down to us. He was Aerius, who about the middle of the fourth century became the leader of a small Puritan party within the Church. He had the direction of an establishment at Sebaste, in Pontus, which offered accommodation to both strangers and the poor. The first germ of the Hospital idea meets us towards the close of the second century. At about this date, in times of war, there were attached to the Roman camps a "valetudinarium" and a "veterinarium." In the former, only those were treated who were seriously wounded or diseased: previously to this time the disabled soldier would receive such rough care as the age afforded, either in his own tent or in a neighbouring cottage. Under Tiberias, who paid no little attention to the well-being of his soldiers, ambulances with easy cushions were provided for the transportation of the sick. But the establishment of this valetudinarium was, after all, only a military resource, and it cannot be doubted that the real origin of hospitals is Christian, and to be credited to the Eastern and not to the Western Church. The first complete establishment for the reception of the sick was built by Basil the Great at Cæsarea, in Cappadocia (Asia Minor), soon after he became Archbishop of that see (A.D. 370). Cæsarea at this time was a city of no inconsiderable importance, and the work of St. Basil was so extensive as to acquire the title of "the new city," subsequently also receiving the name of "the Basileiad." It was, in fact, not one building but a group of several. It included a church, with a palace for the bishop and residences for his attendant clergy; houses for the poor, the sick, and travellers, and workshops for the labourers who were needed. There was also a special part devoted exclusively to the lepers, upon whom St. Basil bestowed his personal care, and whom he did not fear to kiss. The head of this city-within-a-city was a Chorepiscopus, a dignitary nearly corresponding to our Suffragan Bishop. A contemporary of St. Basil has left us a brief description of the place. The writer is Gregory Nazianzen, the fellow-countryman and fellow-student of St. Basil, who enjoyed his friendship throughout his life—"Go forth a little from the city and behold the new city, the treasure-house of godliness, in which disease is investigated and sympathy proved. We have no longer to look upon the terrible and pitiable sight of men like corpses before death, with the greater part of their limbs dead." The actual care of the sick devolved, of course, upon the monks whom St. Basil had gathered together, and to whom he gave his celebrated rule, to this day the one chiefly followed in the Greek Church. One curious regulation is worth noticing: "Any nurse giving to a patient any food except that ordered by the Superintendent was to be deprived of benediction." St. Basil himself throughout his career led an extremely ascetic life. He took but one meal a day, not of flesh, but of bread, salt, and herbs. He wore the same threadbare garments night and day, and took his rest upon no softer bed than a plank, enduring all these hardships in spite of the rigors of the climate of Cappadocia, and without remitting his own unceasing activity.

At no great distance came St. Chrysostom (died 407), who is recorded to have expended the superfluous revenues of his see (Constantinople) in the establishment and maintenance of hospitals for the sick. These were ruled by two presbyters, having under them an army of physicians, cooks, and "kind attendants."

Under the Emperor Julian, evil days once more fell upon the Christian Church. However, although Julian endeavoured to supersede Christianity by a kind of Gnostic Paganism, he turned to the faith he hated for the models of the reforms he wished to introduce. It was doubtless as much with the desire of excelling the clergy in their own work as of benefiting his subjects, that Julian decreed the establishment in various places of hospitals for the reception of the sick, and hospices for the entertainment of travellers.

Turning to the west, the early traces of hospitals are by no means clear. That founded by Fabiola at Rome is said to have been the first established in that city. Paulinus of Nola (in Campagna), a friend of St. Jerome and St. Augustine, has left us three lines in one of his poems which describe the hospital he himself had founded—for the reception, however, of the poor and the aged rather than of the sick. Translated, they run thus (the original metre being preserved):—

"Scattered in groups of three on the long rows of hospital benches,

Sad and decrepit, the crowd of poor old men make a clamour,

While in a chattering flock the grey haired mothers foregather."

Jerome also is said to have founded a hospital for the sick at Bethlehem.

Such were the slender beginnings from which have developed those great establishments which have done, and are doing, so much to mitigate human suffering. W. H. W.

